# Assessment of the time taken for blood culture positivity in infectious endocarditis due to Candida and an analysis of the utility of beta-D-glucan in early detection of Candida endocarditis: a multicenter retrospective study

**DOI:** 10.1017/ash.2025.10154

**Published:** 2025-10-03

**Authors:** Farha Karlath, George R. Thompson, Deepak Kumar, Tahereh Shokohi, Richard T. Ellison

**Affiliations:** 1 Department of Medicine, Division of Infectious Diseases & Immunology, UMass Chan Medical School, Worcester, MA, USA; 2 Department of Medicine, Division of Infectious Diseases, UC Davis School of Medicine, Sacramento, CA, USA; 3 Department of Medicine, Division of Infectious Diseases. All India Institute of Medical Sciences, Jodhpur, India; 4 Department of Mycology, Mazandaran University of Medical Sciences, Sari, Iran; 5 Department of Microbiology and Physiology, UMass Chan Medical School, Worcester, MA, USA

Dear Editor,

*Candida* endocarditis is a rare but serious fungal infection that has become an increasing concern in the context of the ongoing opioid epidemic in the United States.^1^ The growing prevalence of intravenous drug use (IVDU), particularly among individuals using opioids, has contributed to a rise in infections associated with injection drug use, including fungal endocarditis. Early diagnosis remains a challenge due to its often-insidious presentation and the difficulty in distinguishing it from other forms of infective endocarditis.^2,3^

We present findings from a multicenter, retrospective study aimed at assessing the time to blood culture positivity in patients with *Candida* endocarditis and evaluating the potential role of ^1,3^-beta-D-glucan (BDG) testing for early detection. We reached out to over 30 investigators with previously published data on *Candida* endocarditis and successfully obtained patient-level datasets from four academic medical centers across three countries. These centers provided a combined cohort of 36 patients diagnosed with Candida endocarditis between January 2010 and June 2024; 26 of whom have previously been reported in the literature.^2,3,4,5^ Diagnosis was confirmed via echocardiography detecting vegetations and blood culture growing Candida.

The mean time from symptom onset to blood culture positivity was 21.8 days, with 50% of patients having at least one prior negative blood culture; presumably due to prolonged time to positivity of fungal blood culture. Empiric antifungals were not given prior to these tests. Risk factors for infection included a history of injection drug use in 72%, the presence of an intravascular catheter in 20%, and total parenteral nutrition in 8%. Additionally, 8% of patients had cardiovascular risk factors, including prosthetic valves or a history of cardiac or mediastinal surgery. *Candida albicans* was the most identified species (44.4%), followed by *C. parapsilosis* (22.2%), and *Nakaseomyces glabratus* (formerly *C. glabrata)* (19.4%). Less common species included *C. tropicalis* and *C. dubliniensis*, each representing 5.6% of the cases, while *C. kefyr* was the least prevalent at 2.8%.

BDG testing was performed in 25% (9/36) of patients, with 78% (7/9) showing elevated levels (>80 pg/mL, range: 150–520 pg/mL, median: 210 pg/mL). Testing was conducted at varying time points across patients, with a turnaround time of 24–36 hours, preceding blood culture positivity by a median of 3 days. In the BDG-tested group, C. albicans (67%, 6/9) predominated. The two patients with non-elevated BDG had grown *Candida parapsilosis* in the blood culture.

Delaying antifungal therapy while awaiting blood culture results has been shown to increase hospital mortality for patients with candidemia.^3,6^ While our study population is small, the findings demonstrate that for *Candida* endocarditis there can be both a relatively prolonged delay between symptom onset and diagnosis, as well as the limitations in detection with current blood culture systems. The finding of BDG positivity in most of the tested patients suggests its potential for earlier detection, especially when blood cultures are negative. This aligns with the 2025 American Heart Association (AHA) guidelines on blood culture-negative endocarditis, where despite a relative lack of supporting data, the guidelines committee recommend BDG as a biomarker for suspected fungal endocarditis to guide early antifungal therapy.^7^

Despite its potential, there are important caveats in the use of BDG as a diagnostic marker. In particular, BDG levels can be influenced by factors such as hemodialysis, prior antifungal therapy, or certain antibiotics like piperacillin-tazobactam, which may result in false-positive results.

Our study has limitations, including its retrospective design and small sample size, and further prospective studies are needed to validate BDG’s role in severe metastatic *Candida* infections, determine optimal cutoff values, and assess its utility across diverse patient populations.

Sincerely,

Farha Ahmed Karlath, MD; Richard T. Ellison III, MD; George R. Thompson III, MD; Deepak Kumar, MD; Tahereh Shokohi, PhD.
